# Supplementary material for: Are carbon emissions trading and green financial instruments synergistic? -Comprehensive quantitative research based on content analysis
Source: PLoS One. 2024 Mar 7;19(3):e0298601. doi: 10.1371/journal.pone.0298601 (PMC10919601; doi:10.1371/journal.pone.0298601)
Supplement: S1 Data — (ZIP) [file pone.0298601.s001.zip › Data/地级市Z_F工作报告.zip!/╦─┤¿/π≥╓▌╩╨/└┤╫╘╣1⁄2╓┌║┼í╛┬φ┐╦╩2╛▌═°í┐_2019.docx]

各位代表：

现在，我代表市人民政府，向大会报告工作，请予审查，并请市政协委员和列席人员提出意见。

2019年工作回顾

2019年，我们深入学习贯彻习近平新时代中国特色社会主义思想、党的十九大和十九届二中、三中、四中全会精神，在省委、省政府和市委的坚强领导下，在市人大、市政协的监督支持下，认真践行新发展理念，扎实推进“六大攻坚行动”，有效应对风险挑战，经济社会保持平稳健康发展。叙永县、古蔺县顺利通过贫困县退出省级专项评估检查，龙马潭区、江阳区分别被表彰为全省县域经济发展强县、进步县（城市主城区），成功获国务院批准设立泸州综合保税区、跨境电子商务综合试验区，创建为全国社会信用体系建设示范城市，列入中央财政支持开展居家和社区养老服务改革试点地区。全市地区生产总值突破2000亿元大关，达到2081.3亿元、总量居全省第6位，增长8%、增速居全省第4位。

一年来，我们扎实开展“不忘初心、牢记使命”主题教育，政府自身建设不断加强。始终把政治建设摆在首位，认真贯彻中央和省委、市委决策部署，扎实做好学习教育、调查研究、检视问题和整改落实，推动“不忘初心、牢记使命”主题教育往深里走、往心里走、往实里走，形成了一批具有时代特征和泸州特色的理论成果和实践成果。结合实际开展“走进群众、服务群众、引导群众”等活动，一批涉及群众切身利益的问题得到有效解决。深入推进“放管服”改革，在全省率先开展“0证明城市”创建，政务服务持续优化，深化商事制度改革获国务院办公厅通报表扬。全面推进全国法治政府建设示范市创建，严格执行市人大及其常委会决议决定，自觉接受市政协民主监督，办理人大代表建议134件、政协委员提案267件，法定时限办复率100%。切实加强地方政府立法，起草地方性法规草案1部，制定出台政府规章2部。深入推进政府系统党的建设，切实严明政治纪律、政治规矩，大力整治形式主义、官僚主义和干部队伍中存在的“五种不良情绪”，着力强化审计监督，纵深推进党风廉政建设和反腐败斗争，政府系统执行力、战斗力、凝聚力进一步增强。

一年来，我们着力推动产业升级，经济实力明显增强。全年规模以上工业增加值增长10%；全社会固定资产投资增长11.9%；社会消费品零售总额851亿元，增长11.4%；地方一般公共预算收入159.6亿元，同口径增长13.2%，获得省转贷地方政府债券114.8亿元。传统产业提质发展，泸州老窖酿酒工程技改项目投粮试生产，郎酒泸州兼香基地项目加快建设，白酒税收占全市税收的30%，酒业优势进一步凸显；聚碳酸酯项目（一期）全面达产，“四大产业”增加值增长8.6%。电子信息、装备制造、新能源新材料、现代医药、航空航天等新兴产业集聚发展，驰腾科技、长机公司等企业产品远销海外，容大智能CVT变速箱、中航转向系统等实现量产，西南无人机项目、维思达生产基地建成投产，鑫阳钢铁技改项目开工建设，成功引进恒力（泸州）智能化新材料产业园项目。获批省级开发区6个，全市园区营业收入突破3050亿元、增长16%。现代服务业加快发展，古郎景区创建为国家4A级旅游景区，合江尧坝镇、古蔺太平镇入选四川省第一批文旅特色小镇，全市实现旅游总收入596亿元、增长16.2%；引进金融机构3家，银行业金融机构各项贷款余额增长16.3%；宏图物流成功创建国家5A级物流企业。现代农业稳步发展，建成标准化产业基地11万亩，粮食生产连续8年稳产，合江带绿荔枝入选全国十大特异资源品种，纳溪区创建为国家农村产业融合发展示范园，泸县创建为国家农业绿色发展先行区。

一年来，我们着力深化改革扩大开放，发展活力更加彰显。自贸试验区平台不断升级，新形成制度创新成果80项，“生产企业出口退税服务前置”经验全国推广，进口肉类指定监管场地建成投用，进境粮食中转量突破15万吨，泸州港外贸箱量实现4.8万标箱、增长33%。开放通道不断畅通，列入《西部陆海新通道总体规划》北部湾出海口主通道，川南城际铁路加快建设，渝昆高铁四川段启动建设，成功增设泸州东站；江习古高速（古习段）、G8515泸荣高速建成通车；云龙机场开通航线38条，年旅客吞吐量达186万人次；全国综合运输服务示范城市创建扎实推进，泸州港多式联运示范工程通过国家验收。开放水平不断提升，与德国黑尔讷、爱尔兰塔拉莫尔结成友好城市，到位市外国内项目资金480亿元。重点领域改革加快推进，泸州老窖集团、港投集团等国有企业下属子公司混合所有制改革有序推进，泸天化集团完成司法重整，中海沥青（四川）有限公司完成混改。乡镇行政区划调整改革稳步推进。农村集体资产清产核资全面完成。获批组建国家技术转移西南中心泸州分中心，泸州国家高新区列入国家知识产权试点园区，医药产业园被科技部确定为国家级科技企业孵化器。资源枯竭型城市转型发展获国务院办公厅通报表扬。

一年来，我们着力提升城市品质，城乡面貌持续改善。两江新城加快建设，沱江新城整体城镇化项目、健康科技城、第二医教园区加快推进，佳乐世纪城下穿隧道竣工通车，长江滨江路小关门至柏木溪段、长江六桥北连接线达到通车条件，长江二桥及连接线工程、二环路全段加快建设。建设海绵城市10平方公里。累计建成乡镇污水处理厂128座，在全省率先实现乡镇污水处理厂全覆盖；累计新改建城镇污水管网560公里。城市生活垃圾无害化处理率达100%。建成开放城市公园3个，新增城市绿地380公顷。实施城镇危旧房棚户区改造677套。顺利通过全国文明城市复检。江阳区分水岭镇创建为省级特色小城镇，新增四川省“百镇建设行动”试点镇5个，建成省级“宜居乡村”280个。

一年来，我们着力推进精准扶贫，整体脱贫顺利实现。整合各类扶贫资金63.86亿元，实施项目132个，建设脱贫产业园334个，就业类扶贫基地、扶贫车间44个，累计转移就业贫困劳动力10.3万人。鸡鸣三省大桥等5座渡改桥全面建成，赤水河环线扶贫公路古蔺段建成通车，新改建农村公路1608公里，实现所有建制村通硬化路、通客运。解决2530名贫困群众饮水安全问题。贫困村电网升级改造、卫生室建设全面完成。实现贫困县达标中心校、达标卫生院乡镇全覆盖。实施农村危房改造8095户。10.1万名建档立卡贫困人口纳入兜底保障。全年实现2.8万人脱贫、14个贫困村退出。

一年来，我们着力夯实生态基底，环境问题有效整治。坚决打好污染防治“八大战役”，狠抓环境突出问题整治，加快推进长江生态保护修复，中央和省级生态环保督察反馈问题整改率90%，罗汉街道老鹰坵市政污水溢流口整治得到国家推长办肯定。主城区空气质量优良天数达306天。扎实推进河（湖）长制，地表水国控考核断面水质优良率85.71%，长江出川断面水质保持Ⅱ类标准，县级及以上城市集中式饮用水水源水质达标率保持100%，顺利通过全国人大常委会《水污染防治法》执法检查，成功承办2019年中国赤水河流域生态文明建设协作推进会。综合治理岩溶石漠化50平方公里，水土流失综合治理201.5平方公里，全市森林覆盖率达50.8%，土壤环境质量总体良好。成功承办“天府使命—2019年长江出川断面突发环境事件应急演练”，得到生态环境部充分肯定。

一年来，我们着力补齐民生短板，民生福祉不断提升。民生支出占一般公共预算支出比重达70.3%。预计实现城镇居民人均可支配收入37252元，增长9.1%；农村居民人均可支配收入16531元，增长10.3%。城镇新增就业4.67万人，城镇登记失业率2.25%。城乡居民医保参保率98%。农民工服务保障工作走在全省前列。新建公办幼儿园25所。“全面改薄”工程顺利完成，开工建设教师周转房1007套，招收公费定向培养村小教师184名。江阳城建职业学院、泸州高中城西校区（一期）建成投用，泸州医疗器械职业学院列入省“十三五”高校设置规划。全民预防保健免费体检177万人次，市中医院城南医院、市妇幼保健院（二期）等项目主体完工，西南医疗康健中心（一期）、市残疾人康复中心建成投用。泸县、合江县列入全国紧密型县域医共体试点。全国养老服务业综合改革试点深入开展，7.9万名困难老人享受居家养老服务，适老化改造养老机构床位2147张。新增全国重点文物保护单位6处，泸县创建为第一批四川省现代公共文化服务体系示范县。成功举办ITF国际网球公开赛。统筹推进防灾减灾救灾工作，严格落实安全生产责任制，安全生产重大风险防控能力明显提升，全市安全生产形势总体稳定，泸县、合江县创建省级食品安全示范县通过验收。获评全国“七五”普法中期先进城市。全国社会治安防控体系标准化城市建设扎实开展，扫黑除恶专项斗争纵深推进。纳溪区“慧眼工程”得到中央领导同志肯定，龙透关派出所被命名为全国首批“枫桥式公安派出所”，摩尼人民法庭被国务院表彰为全国民族团结进步模范集体。

高质量完成全国第四次经济普查和第三次国土调查。国防动员、民兵预备役、人防工作切实加强，档案、保密、气象、地方志、社会科学工作顺利推进，援藏援彝、民族宗教、外事、侨务、对台、妇女儿童、青少年、双拥、老龄、慈善、残疾人工作取得新成绩。

各位代表，过去一年，我们面临多年来少有的严峻形势和困难挑战，全市经济社会发展取得的成绩来之不易，这是省委、省政府和市委坚强领导的结果，是市人大、市政协监督支持的结果，是全市广大干部群众齐心协力、共同奋斗的结果。在此，我代表市人民政府，向各位人大代表、政协委员，并通过你们向全市各族人民、驻泸部队、武警官兵、政法干警、各民主党派、工商联、无党派人士、各人民团体和各界朋友，表示衷心感谢并致以崇高敬意！

同时，我们也清醒地看到，政府工作还存在许多不足，发展还面临不少困难。主要表现在：经济下行压力加大，发展新动能有待加快培育；外向型经济发展程度不高，开放水平与先进城市还有较大差距；脱贫攻坚与乡村振兴的有机衔接不足，教育、医疗、养老、住房等民生保障存在薄弱环节；生态环保、安全稳定任务仍然繁重，基层治理能力需要进一步增强；部分政府工作人员践行新发展理念、推动高质量发展的水平有待进一步提升。对此，我们将直面问题、勇于担当，采取有效措施切实加以解决。

2020年工作安排

2020年是全面建成小康社会和“十三五”规划收官之年，也是市委确定的“开放合作、产业突破”之年。我们要始终坚持以习近平新时代中国特色社会主义思想为科学指引，深入贯彻党的十九大和十九届二中、三中、四中全会精神，抢抓“一带一路”和长江经济带、成渝地区双城经济圈建设等国家重大战略机遇，深入贯彻省委“一干多支、五区协同”“四向拓展、全域开放”决策部署，全面落实市委八届八次全会精神，紧扣全面建成小康社会目标任务，坚持稳中求进工作总基调，坚持新发展理念，坚持以供给侧结构性改革为主线，坚持以改革开放为动力，推动高质量发展，坚决打赢三大攻坚战，深入实施“六大攻坚行动”，统筹抓好稳增长、促改革、调结构、惠民生、防风险、保稳定各项工作，确保全面建成小康社会和“十三五”规划圆满收官，科学谋划“十四五”发展，奋力朝着全省经济副中心、成渝地区双城经济圈重要节点城市和川渝滇黔结合部区域中心城市目标迈进。

经济社会发展的主要预期目标是：地区生产总值比2019年（下同）增长8.5%左右，规模以上工业增加值增长9.5%，全社会固定资产投资增长9%，社会消费品零售总额增长10%，地方一般公共预算收入同口径增长9.5%，城镇居民人均可支配收入增长8%以上，农村居民人均可支配收入增长9%以上。

一、坚持优势优先，推动白酒产业高质量发展

着力做大规模。抢抓白酒产业政策调整新机遇，充分发挥优质资源和产区优势，积极引入业外资本、业内知名企业，加快构建“一核三带”酒业发展格局。支持泸州老窖、郎酒、川酒集团三大龙头企业做优做强做大，支持郎酒加快上市步伐，推动酒类企业抱团集聚发展；支持3朵“川酒小金花”、26户“小巨人”企业提质发展。确保泸州老窖酿酒工程技改、川酒集团永乐基地改造等项目投产达产，加快推进泸州老窖智能化包装中心技改、郎酒吴家沟生产基地续建等项目，开工建设老窖集团智能仓储物流、玉蝉酒业胡市生产基地等项目，启动茅溪酱酒产业园建设。全市白酒营业收入突破1000亿元、增长10%，泸州白酒产业园区产值和服务性收入突破1000亿元、增长17%。

着力做优品质。强化产区生态保护，坚持纯粮固态酿造，加快建设专业化、规模化、产业化酿酒原粮基地。强化老窖池资源保护、整合和综合利用，持续优化高端产能结构。大力弘扬“工匠精神”，加强酿酒、品酒、勾调等高技能型人才培育，确保传统酿造技艺世代相传。加大科研投入与成果转化应用，着力提升国家固态酿造工程技术研究中心、国家酒类及加工食品质量监督检验中心、国家酒类包装产品质量监督检验中心功能，加快推进原酒质量标准化体系建设，提升白酒品质。

着力做响品牌。突出国窖1573、青花郎引领作用，支持泸州老窖“五大单品”、郎酒“一树三花”品牌持续提升；支持川酒集团加快品牌整合壮大，推动泸州原酒品牌化、标准化建设；鼓励泸酒企业加快新品开发，加强泸酒知识产权保护，建设优质白酒品牌集聚区。高质量办好第十五届中国国际酒业博览会、“泸酒丝路行”等系列活动，提升泸酒品牌国际影响力，助力泸酒企业“走出去”。做强白酒制造、包装材料两大产业链，创新“白酒+文旅”“白酒+金融”等业态，加快推进郎酒庄园、酒镇酒庄等特色发展，高质量打造“中国白酒·泸州产区”。

二、坚持调优结构，加快制造业转型升级

加快壮大电子信息产业。大力发展智能终端产业，积极引进研发设计、关键零部件制造、整机制造、供应链金融等企业，支持明德亨电子、华景光电、天诺科技等一批重点企业做强做大，加快液晶光电与人工智能产品研发制造中心等项目建设，推动中国电子（泸州）产业园落地见效，加快构建电子信息产业生态圈和创新生态链。大力发展数字经济，着力推进数字产业化，加快西南（泸州）容灾备份中心等重点项目建设，推动5G网络、IPv6规模部署，创新打造一批“5G+”行业应用示范工程，创建国家新型工业化产业示范基地（大数据）。

振兴发展装备制造产业。大力发展汽车及零部件、工程机械及液压元件、油气钻采装备等产业，确保泸州豪能汽车同步器系统智能生产基地、泸州智通自动化酿酒设备研发生产线、海科机械高端装备技改扩能、中信重工特种智能机器人生产基地等项目建成达产，推动智能装备在酿酒、机械、汽车零部件、电子信息等行业普及应用，打造一批智能制造示范工厂，加快构建具有竞争优势的装备制造产业体系。

转型做精能源化工产业。加快推进页岩气勘探开发和综合利用。依托化工基础优势，推动化工产业向精细化工和化工新材料方向发展，提升产业科技含量和产品附加值。支持泸天化、北方化工、合盛硅业等加大科研投入，研发生产聚碳酸酯、纤维素醚、有机硅等下游产品。力争恒力（泸州）智能化新材料产业园（一期）建成投产。

加快发展现代医药产业。依托医药产业园，发挥国家级科技企业孵化器、西南医科大学成果转化基地、川渝医药行业协同创新战略联盟等平台功能，大力开展补链强链招商和科研成果孵化，着力构建中药、化学药、生物制药、医疗器械、医药衍生和配套产业五大产业链。启动建设康复辅助器具特色产业园，加快步长生物制药基地及新药产业化基地、科瑞德研究院、金佳钲医疗器械生产基地等项目建设，打造医药研发及产业化生态集群。

培育发展航空航天产业。务实推进航天科工集团三十一研究所空天动力项目、北航泸州研究院项目、中航工业601所无人机项目建设。加快科技成果转化，强化以产哺研，着力打造集研发、制造、销售及人才培养为一体的国家级空天动力研制基地、中小型发动机生产基地和无人机产业基地。

三、坚持突出特色重点突破，繁荣发展现代服务业

壮大服务业支柱。大力发展商贸服务，改造提升主城区成熟商圈，推动西南商贸城提档升级，力争万诚国际中心（三期）、欢乐汇（二期）等项目竣工投用，打造夜间消费集聚区，推动消费扩容提质。完善长效管理调控机制，促进房地产市场平稳健康发展。大力发展现代金融，力争新引进金融机构2家以上，银行业金融机构各项贷款余额增长12%以上。大力发展文化旅游，规划建设长征国家文化公园（泸州段），加快中国酒城·长江生态旅游带、华商同心文化园、罗汉林小镇等项目建设，推动黄荆老林、福宝景区、天仙硐景区等提档升级，鼓励争创天府旅游名县。深入推进农商文体旅融合发展，积极创建一批特色文旅品牌。力争全市旅游总收入660亿元，增长10.7%。

打造服务业特色。聚焦建设区域医疗健康中心，支持域内医疗机构加快发展，鼓励建立跨区域医联体或专科联盟，进一步提升区域医疗服务能力。深入推进全国居家和社区养老服务改革试点，强化医养结合，推动医疗医药、养生养老、健康体育等多业态深度融合发展。聚焦建设区域教育培训中心，深化产教融合、校企合作，加快发展多种形式的教育培训服务；推动高校内涵式发展，支持西南医科大学建成博士学位授予单位（点），支持高校面向“一带一路”、长江经济带和川渝滇黔区域培养专业技术人才。大力发展会展经济，创新办好中国（泸州）西南商品博览会、农产品交易博览会和国际城市会展论坛等系列活动。

培育服务业高端。推动大数据与其他产业融合发展，大力发展软件信息服务业，开展数据储存、数据价值挖掘、数据分析应用等“大数据+”服务，推进区块链技术应用，拓展数据增值应用，争创全国特色型信息消费示范城市。加快推进白酒产业计量测试中心建设，搭建一批融合检验检测、分析试验、技术研发等功能的服务平台。大力发展工业设计、文化创意、科技服务、健康护理等服务外包，推动产业链向高端延伸。

四、坚持以自贸区、综保区平台为引领，推进全方位高水平对外开放

着力试制度拓平台。全面完成自贸区《总体方案》试验任务。突出“海关、海事、金融、税务、政务服务”等领域制度创新，扎实开展“证照分离”改革全覆盖试点，确保形成创新成果50项以上。围绕“保税加工制造、保税研发设计、保税物流分拨、保税检测维修、保税销售服务”五大中心功能，加快综合保税区基础设施和监管设施建设，大力招引保税加工、保税物流、保税服务企业，确保通过国家验收。全力推动云龙机场临时开放口岸申创工作。完善进境粮食指定口岸、进口肉类指定监管场地、跨境电商综试区等平台功能，确保泸州港外贸箱量突破5万标箱、进境粮食中转量18万吨、进口肉类中转量5000吨，实现保税进出口额20亿元，带动全市实现外贸进出口总额80亿元。

着力畅通道快物流。深化港口型国家物流枢纽承载城市建设，加快建设长江上游（四川）航运中心，规划建设“四港联动”国际物流中心，全力争创全国城乡高效配送试点城市。积极推动西部陆海新通道建设，力争川南城际铁路正线、站房主体基本建成，加快推进渝昆高铁泸州段、隆黄铁路叙毕段建设，力争隆黄铁路隆叙段扩能改造开工，争取蓉遵高铁、泸遵货运铁路列入国省铁路网规划。加快泸永高速、白沙长江大桥、河东长江大桥等项目建设，开工建设古金高速、榕山长江大桥、美酒河特大桥等项目，确保叙威高速形成通车能力。云龙机场国内通航城市达40个以上，完成年旅客吞吐量220万人次。

着力强贸易促合作。积极培育跨境电商、平行进口汽车等贸易业态，加快建设临港进出口加工中心、商品集散中心、大宗商品交易中心。深化与长江沿线港口城市合作，优化长江升船机外贸直航班轮开行方式，扩大启运港退税覆盖范围。加快推进川南经济区一体化，进一步优化泸州至粤港澳地区、北部湾地区陆海联运通道。加强同青白江国际铁路港合作，提升“蓉欧+泸州港”班列运营效能。

着力兴产业增后劲。积极承接长三角、京津冀、粤港澳大湾区优势产业转移，围绕航运物流、先进制造、现代服务业等涉水涉港涉外产业，全力推进产业链招商，全年招引5亿元以上项目40个，实际到位市外国内项目资金520亿元以上。支持综合保税区关联产业发展，推动保税加工、保税物流等产业发展壮大，支持和润粮油、厦门建发等企业加快发展，做大做强以粮油、塑料粒子、基础化工品等为核心的大宗商品交易规模。

五、坚持人民城市为人民，加快建设川渝滇黔结合部区域中心城市

持续优化城市格局。积极融入成渝地区双城经济圈建设等国家重大战略，主动联合重庆毗邻区域，加快推进川渝融合发展先行试验区规划建设，以轨道交通引领城市格局，积极推进区域规划、基础设施、产业布局、生态环境、体制机制、公共服务等一体化进程，努力为一域增光、为全域添彩。将“公园城市”理念贯穿城市发展全过程，统筹考虑自然山水布局、城市功能分布和土地价值，着力优化“三生”空间，科学确定城市开发强度。完成国土空间总体规划编制，初步形成国土空间开发保护一张图。加快城西新城、高铁新城、茜草半岛、长江湿地新城建设，推进城市组团式发展。支持县城特色化发展，做强做大中心镇、特色小镇，新创建省级“百镇建设行动”试点镇5个。

加快完善城市功能。确保二环路纳溪段竣工投用，全力推进长江六桥、长江二桥建设，扎实做好长江五桥前期工作，健全城市路网骨架。统筹推进智慧城市和公共交通网络等基础设施建设，建设海绵城市6平方公里。着力优化产业园区布局，推动产业园区向产业功能区转变，打造以人为中心、主导产业十分鲜明、宜业宜居的新城组团。按照“公共、共享”的原则，科学布局教育、医疗卫生、文化体育等公共资源，力争完成四川化工职业技术学院整体搬迁项目建设，开工建设西南医疗康健中心（二期）、西南医科大学附属口腔医院新院、市疾病预防控制中心，确保市中心血站整体完工。推进城市园林绿化提档升级，加快酒谷湖、渔子溪、柏木溪等城市公园建设，持续打造“两江四岸”百里绿色长廊，着力构建城市绿色生态体系。

创新推进城市治理。巩固提升全国文明城市创建成果，认真落实“街长制”，加快建设示范化邻里中心，打造标准化示范街区。建成城北、城西垃圾压缩中转站，中心城区生活垃圾分类覆盖率达50%。启动城东污水处理厂（二期）、城南污水处理厂（二期）、纳溪污水处理厂（三期）建设，全面完成主城区雨污分流改造。探索“城市大脑”建设，积极推进智慧停车、智慧执法、智慧市政一体化，推动城市治理科学化、智慧化、精细化。

六、坚持生态优先，推进长江及其支流绿色发展

打好污染防治攻坚战。突出精准治污、科学治污、依法治污，提升污染防治能力。强化大气污染综合治理，确保主城区空气质量优良天数达78.9%以上。深入落实河（湖）长制，扎实开展龙溪河、濑溪河、大树河流域环境整治，确保全市地表水国控考核断面水质优良率达85.71%以上。加快省级土壤环境风险管控试点区建设，推进土壤污染治理修复试点，确保土壤环境质量总体稳定。

加强生态保护修复。落实“三线一单”，强化生态分区管控，严格执行企业排污许可、环境信用评价制度。实施最严格的耕地保护和水资源管理制度，提高资源利用综合效益。加强自然保护地、饮用水水源保护区等生态敏感区管控。深入实施绿化泸州行动，完成营造林30万亩。推进废弃工矿地等受损毁自然生态系统恢复重建，综合治理岩溶石漠化50平方公里，水土流失综合治理140平方公里。

加快推动绿色发展。推进园区循环化改造，加快大洲化工、泸州富邦等工业企业退城入园步伐，培育壮大节能环保产业，推进建材、化工等传统产业绿色化改造，确保完成单位GDP能耗下降目标任务。加快发展生态农业、生态旅游、生态康养等绿色产业，推进林竹产品配套精深加工。加强生态文明宣传教育，推动形成简约适度、绿色低碳的生活方式。加快建设长江经济带高质量发展先行区，着力讲好新时代绿色发展“泸州故事”。

七、坚持城乡融合发展，持续提高群众幸福指数

决战决胜脱贫攻坚。扎实抓好剩余3618名贫困人口脱贫工作，确保全面完成脱贫攻坚任务。严格落实“四个不摘”总体要求，持续深化产业扶贫、就业扶贫、教育扶贫、健康扶贫，加快贫困地区基础设施建设，做好易地扶贫搬迁后续工作，稳定实现“两不愁三保障”。全面开展脱贫攻坚普查，强化脱贫监管，保证脱贫质量。及时做好返贫人口和新发生贫困人口帮扶，探索建立解决相对贫困的体制机制，提升贫困群众自我发展能力。

深入推进乡村振兴。推动脱贫攻坚与乡村振兴有机衔接，积极争创一批省级乡村振兴先进区县、先进乡镇、示范村。持续深化农业供给侧结构性改革，推动八大特色农业产业提档升级，争创一批省级现代农业示范园区。全面推进电子商务进农村。稳定粮食生产，加强“菜篮子”工程建设，加快恢复生猪生产。切实抓好农民工服务保障，稳定农民工就业，推动农民工返乡创业，多渠道促进农民持续增收。全力推进农村人居环境整治，改造农村卫生厕所3.16万户，30%以上农村聚居点建成污水处理设施，70%以上村民小组配备保洁员，争创省级“宜居乡村”200个，探索推动农业变景观、农村变景区、农居变景点。

大力发展县域经济。坚持因地制宜、特色发展，支持争创全省县域经济发展强县、先进县、进步县。全面落实国家关于民营企业、小微企业扶持政策和四川省“民营经济20条”，引导民营经济规范健康发展，力争民间投资占全社会固定资产投资比重48%以上。做好乡镇行政区划调整改革“后半篇”文章，激发县域经济发展活力。

持续保障和改善民生。优先发展教育事业，推动学前教育普惠发展，新建公办幼儿园19所，普惠性幼儿园覆盖率达80%；基本消除义务教育大班额，确保叙永、古蔺义务教育发展基本均衡县通过国检；加快泸州高中城西校区（二期）、古蔺金兰高中新建工程等项目建设，确保高中阶段毛入学率达90%以上。积极争创全国健康城市示范市，加快紧密型医共体建设，传承创新发展中医药事业，提升全民预防保健服务水平。推进国家医保基金监管方式创新试点。全面实施药品降价改革。繁荣发展文体等社会事业，推进公共文化服务标准化建设，抓好“五馆”免费开放，办好泸州市第八届运动会、第七届残疾人运动会。鼓励创业带动就业，城镇新增就业3.65万人。深入实施全民参保计划，推进养老保险全覆盖。深化社会救助兜底保障改革。扎实做好退役军人工作，深入开展“全国双拥模范城”续创。支持国防和军队改革，加强国防动员和后备力量建设。开展第七次全国人口普查。积极发展妇女儿童、青少年、老龄事业，切实做好人民防空、民族宗教、外事、侨务、对台、保密、档案、地方志、社会科学等工作。

八、坚持深化改革创新，让发展更具动力更有活力

持续优化营商环境。深入推进“放管服”改革，全面落实“非禁即入”要求，严格执行市场准入负面清单，加快公共数据资源共享应用，巩固提升“0证明城市”创建成果，推进“双随机一公开”全覆盖，开展政务服务“好差评”。全面落实减税降费政策，进一步降低企业税费、要素、用工、物流成本，助力实体经济做大做强。深化社会信用体系建设，积极探索信用建设服务实体经济发展新路径。

深化重点领域改革。深入推进农村集体产权制度改革，探索集体经济发展有效实现形式。深化农村土地制度改革，推进农村资产资源“三权分置”。深化财税体制改革，全面实施预算绩效管理，优化财政支出结构，扎实推进增值税、消费税改革。继续推进市属国有企业混合所有制改革，稳妥推进市属国有平台公司优化重组，积极推动平台公司向城市综合运营商转型。

加强创新主体培育。加快建设国家知识产权试点城市和省级创新型城市，促进“大众创业、万众创新”。支持航空航天产业园区申创省级高新技术产业园区、西南医科大学申创国家大学科技园区。推进创新平台建设，积极争创省级工程技术研究中心、科技成果转移转化示范区、技术转移示范机构。推动川大泸州产研院、西南财大泸州自贸区研究院建设，加快高新技术企业培育和重大科技成果转化，培育省级科技成果转移转化示范企业10户以上。

九、坚持共建共治共享，建设“平安泸州”升级版

着力防范化解风险。加大银行不良贷款处置，规范整治互联网金融，严格政府债务管理，牢牢守住不发生系统性区域性风险底线。深入推进安全生产清单制管理，加强重点行业领域安全风险排查和专项整治，坚决遏制重特大安全事故发生。加强应急管理体系和能力建设，切实强化防汛抗旱、地灾防治和水上应急救援等工作。落实“四个最严”要求，扎实推进国家食品安全示范城市创建。

切实加强依法治理。加快法治泸州建设，深入推进“法律八进”。坚持和发展新时代“枫桥经验”，健全完善公共法律服务体系，深化人民调解、行政调解、司法调解、检察调解衔接机制，构建诉调、访调、警调、检调联动的矛盾纠纷多元化解体系。畅通群众诉求表达渠道，依法及时有效化解矛盾，切实维护群众合法权益。加快全国社会治安防控体系建设标准化（示范）城市创建，深入推进扫黑除恶专项斗争，坚决打击黄赌毒、盗抢骗等违法犯罪行为，进一步增强群众安全感。

构建基层社会治理新格局。着力推进城乡基层治理制度创新和能力建设，健全基层党组织领导的自治、法治、德治相结合的基层治理体系。强化源头治理和网格化管理服务，推动社会治理重心下移、力量下沉、保障下倾。充分发挥自治章程、村规民约在基层治理中的积极作用，拓宽群众参与社会治理的形式和渠道。推进“慧眼工程”“云墙工程”建设和“数字安居社区”试点，提升基层社区治理和服务能力，为城乡居民提供精准化、精细化服务。

各位代表！新时代、新形势、新任务，对加强政府自身建设提出了新的更高要求。我们将坚持以政治建设为统领，以法治建设为保障，以作风建设为抓手，务实转变政府职能，提升依法履职能力水平，着力建设新时代人民满意的服务型政府。加强政治建设。坚决把党的领导落实到政府工作全领域全方位全过程，时刻“不忘初心、牢记使命”，增强“四个意识”、坚定“四个自信”、做到“两个维护”。坚持依法行政。严格执行市人大及其常委会决议决定，自觉接受市人大、市政协监督以及司法监督、审计监督、群众监督、舆论监督；持续推进全国法治政府示范市建设，规范重大行政决策程序，提高行政执法能力水平，全面深化政务公开，夯实基层依法行政基础，确保政府各项工作在法治轨道上运行。提升履职能力。深入践行新发展理念，激励政府系统干部加强思想淬炼、政治历练、实践锻炼，不断提高专业能力、专业素养、专业精神。保持清正廉洁。深入推进政府系统党风廉政建设和反腐败斗争，驰而不息整治“四风”，引导政府系统干部筑牢拒腐防变的思想防线，永葆为民务实清廉的政治本色。

各位代表！全面小康就在眼前，人民群众充满期待。让我们更加紧密地团结在以习近平同志为核心的党中央周围，在省委、省政府和市委的坚强领导下，解放思想、守正创新，鼓足干劲、务实作为，为把泸州建设成为全省经济副中心、成渝地区双城经济圈重要节点城市和川渝滇黔结合部区域中心城市而不懈奋斗！
